# Maternal supplementation with small-quantity lipid-based nutrient supplements during pregnancy and lactation does not reduce depressive symptoms at 6 months postpartum in Ghanaian women: a randomized controlled trial

**DOI:** 10.1007/s00737-017-0752-7

**Published:** 2017-07-11

**Authors:** Harriet Okronipa, Seth Adu-Afarwuah, Anna Lartey, Per Ashorn, Stephen A. Vosti, Rebecca R. Young, Kathryn G. Dewey

**Affiliations:** 1 Program in International and Community Nutrition, Department of Nutrition, University of California, 3135 Meyer Hall, One Shields Avenue, Davis, CA 95616 USA; 2 Department of Nutrition and Food Science, University of Ghana, Legon, Accra, Ghana; 3 Center for Child Health Research, University of Tampere School of Medicine and Tampere University Hospital, Tampere, Finland; 4 Department of Agricultural and Resource Economics, University of California, Davis, CA USA

**Keywords:** Lipid-based nutrient supplements, Postpartum depression, Ghana

## Abstract

We examined the impact on depression at 6 months postpartum of maternal supplementation with small-quantity lipid-based nutrient supplement (SQ-LNS) compared to supplementation with iron and folic acid (IFA) or multiple micronutrients (MMN). In this partially double-blinded randomized controlled trial, pregnant women ≤20 weeks gestation (*n* = 1320) were recruited from antenatal clinics and randomly assigned to receive either (1) SQ-LNS during pregnancy and for 6 months postpartum, or (2) IFA during pregnancy only, or (3) MMN during pregnancy and for 6 months postpartum. Maternal depressive symptoms were measured at 6 months postpartum using the Edinburgh Postnatal Depression Scale (EPDS). Women who scored 12 or more on the EPDS were considered to show symptoms of depression. One thousand one hundred fifty-one women were included in this analysis (LNS = 382, IFA = 387 and MMN = 382). Characteristics of the three groups were similar at baseline, and there were no significant differences between women who were included in the analysis (*n* = 1151) and those who were not (*n* = 169). At 6 months postpartum, 13% of the women overall showed symptoms of depression, and this did not differ by group (LNS = 13.1%, IFA = 11.2% and MMN = 14.7%. *P* = 0.36). The median (25, 75 percentile) EPDS score did not differ by group (LNS 4.0 (1.0, 8.0), IFA 4.0 (1.0, 8.0), MMN 5.0 (2.0, 9.0), *P*_transformed_ = 0.13). Adjustment for covariates did not alter these findings. Maternal supplementation with SQ-LNS compared to MMN or IFA did not affect postnatal depressive symptoms in this sample of Ghanaian women.

## Introduction

Globally, postpartum depression (PPD) has been reported to affect 10–20% of women (Fisher et al. 2012; Nhiwatiwa et al. 1998; O’Hara and Swain 1996; Stewart et al. 2016; Uwakwe 2003). In one study in the Eastern region of Ghana, 9.3% of the women were reported to show symptoms of depression at 6 months postpartum (Okronipa et al. 2012). Over the years, PPD has received attention because of the deleterious effect it may have on both maternal and child health. For example, several studies have reported associations between PPD and increased infant diarrhoeal episodes (Okronipa et al. 2012; Rahman et al. 2007), poor mother-child interactions (Murray et al. 1996) and sub-optimal breastfeeding practices (Henderson et al. 2003).

Inadequate nutrition may play a role in mental health, including depression. Links between nutrient deficiencies and mood have been reported for folate, vitamin B-12, calcium, iron, selenium, zinc and omega-3 fatty acids (Bodnar and Wisner 2005; Leung and Kaplan 2009). Results of randomized controlled trials that have examined nutrient supplementation and PPD have however been mixed. In Guatemala, weekly vs daily supplementation with multiple micronutrients (MMN) for 12 weeks resulted in improved depression scores among women of reproductive age (Nguyen et al. 2009). Among Bangladeshi pregnant women, supplementation with MMN compared to 30 mg iron from 14 weeks gestation to 12 weeks postpartum resulted in lower depression scores (Frith et al. 2009). In Indonesia, however, MMN supplementation from pregnancy to 3 months postpartum had no significant effect on maternal ‘mood’ compared to provision of iron and folic acid (IFA) (Prado et al. 2012). In South Africa, supplementation during lactation with iron (compared to placebo) improved depression among anaemic but not among non-anaemic women (Beard et al. 2005).

Omega-3 fatty acids are another group of nutrients that have received attention because of their potential role in the treatment of depression. Studies in animals have suggested that omega-3 fatty acids can affect brain processes such as those that control mood and anxiety (Carlezon et al. 2005; Delion et al. 1996). However, research in humans on the association between omega-3 fatty acid supplementation and depression has yielded inconsistent results. A meta-analysis of eight trials conducted in patients with depressive symptoms reported a significant clinical benefit of n-3 fatty acid treatment compared to placebo (Grosso et al. 2014). However, another meta-analysis of 12 randomized controlled trials found little evidence to support the use of omega-3 PUFAs to improve depressed mood (Appleton et al. 2006).

Small-quantity lipid-based nutrient supplements (SQ-LNS) were designed to enrich the diets of target groups at high risk of malnutrition (Arimond et al. 2013). They are usually made with vegetable oil, groundnut paste, milk powder, sugar and micronutrients and provide energy, micro- and macronutrients as well as essential fatty acids. We conducted a trial to examine the efficacy of SQ-LNS provided to women during pregnancy and the first 6 months postpartum, and to their offspring from 6 to 18 months of age, on pregnancy outcomes and on child growth at 18 months. As a secondary objective, we sought to examine the impact of fortification of maternal diet with SQ-LNS compared to supplementation with IFA and MMN on maternal PPD. We hypothesized that women who received SQ-LNS during pregnancy would have lower mean Edinburgh Postnatal Depression Scale (EPDS) scores at 6 months postpartum compared to women who received either IFA or MMN.

## Methods

### Study site, design and arms

The International Lipid-Based Nutrient Supplements trial in Ghana (iLiNS-DYAD) was conducted from November 2009 to March 2014 in the lower Manya and Yilo Krobo districts in the Eastern region of Ghana. The area is semi-urban, and is about 70 km from the national capital, Accra. Details of the study design have been described elsewhere (Adu-Afarwuah et al. 2015). Briefly, women were randomly assigned to receive one of the following treatments: (a) daily iron (60 mg) and folic acid (400 μg) capsule during pregnancy, and calcium (Ca) only (akin to a placebo) during the first 6 months postpartum (IFA group); (b) daily multiple micronutrient capsule (1–2 RDAs of 18 vitamins and minerals including 20 mg iron) during pregnancy and the first 6 months postpartum (MMN group); and (c) daily 20 g SQ-LNS during pregnancy and the first 6 months postpartum (LNS group). The 20 g SQ-LNS contained similar vitamin and mineral content as the MMN capsule, plus another four minerals (Ca, P, K, Mg) and essential fatty acids (4.59 g linoleic acid, 0.59 g α-linolenic acid), and provided 118 kcal energy (Table 1). Details of how participants were randomly assigned to the treatment groups have been described elsewhere (Adu-Afarwuah et al. 2015, 2016). The IFA/MMN were provided as capsules in a blister pack whereas the SQ-LNS supplement was in a 20-g sachet.

In a previous paper, we reported a temporary mislabeling of IFA and MMN capsules (Adu-Afarwuah et al. 2015, 2016). Briefly, some women (*n* = 170) who had been initially assigned to the IFA group received the MMN capsule either throughout pregnancy (*n* = 85) or during part of pregnancy (*n* = 85), before receiving the intended IFA capsule the rest of follow-up, and another 170 women initially assigned to the MMN group also received the IFA capsule either throughout pregnancy (*n* = 78) or during part of pregnancy (*n* = 92), before receiving the intended MMN capsule. Women in the LNS group received the correct supplement throughout the study.

In this current analysis, we included all of the women enrolled into the study without discarding any data from those who received the unintended supplements, since the unintended exposure occurred only in the pre-natal period. As previously reported, women in the IFA and MMN groups were exposed to the unintended supplement for only 13% of the follow-up days (Adu-Afarwuah et al. 2016). Our main analysis was based on the supplement women were intended to receive when they were enrolled. In order to address the protocol violation associated with the consumption of mislabeled capsules by some of the women during pregnancy, we conducted two sensitivity analyses; the first examined data based on the supplement that women actually received when they were enrolled, and the second examined data only for women who received supplements after the mislabeling of IFA and MMN supplements had been corrected.

### Recruitment and follow-up

A detailed account of the recruitment process has been given elsewhere (Adu-Afarwuah et al. 2015). Briefly, pregnant women were recruited from the antenatal clinics of two hospitals, one polyclinic and one health center in the study area. Women were considered eligible for the study if they were (1) pregnant, (2) 18 years or older, (3) 20 gestational weeks or less and (4) had an antenatal card complete with history and examination. Women were excluded from the study if they (1) reported milk or peanut allergy, (2) lived outside the study area, (3) intended to move out of the study area within the next 2 years, (4) were unwilling to receive field workers in their homes or take study supplements, (5) were participating in another clinical trial or (6) their antenatal card indicated that they had HIV infection, asthma, epilepsy, tuberculosis or any malignancy. Eligible women were followed up through 6 months postpartum. Supplements were delivered to women bi-weekly during pregnancy and lactation, and information on supplement intake and other data were also collected bi-weekly. The depression questionnaire was administered at 6 months postpartum during a scheduled home visit.

### Outcome variables

Maternal report of depressive symptoms was assessed at 6 months postpartum using the EPDS (Cox et al. 1987). The questionnaire was read aloud to the participant in their own language, and as much as possible, the interview was conducted in private, with only the field worker and participant present. The EPDS is a 10-item scale that documents depressive symptoms that occurred over the past 7 days. Each item was scored on a four-point rating scale that represented the level of occurrence (zero the lowest level of occurrence, three the highest level of occurrence). The scores for all 10 items were then summed up to obtain a total depression score. Women with a total EPDS score of 12 and above were classified as showing symptoms of PPD whereas women who scored below 12 were classified as not showing symptoms of PPD (Chibanda et al. 2010; Lawrie et al. 1998). This questionnaire has previously been used in Ghana (Okronipa et al. 2012) and other African countries (Nhiwatiwa et al. 1998; Stewart et al. 2016; Uwakwe 2003) to measure symptoms of PPD.

### Other variables

We collected other data on maternal and household baseline variables including maternal years of formal education, age, body mass index (BMI, kg/m^2^), marital status, anaemia, gestational age, primiparity, household asset index, household food insecurity index, housing index and season when depression data were collected (wet/dry).

### Statistical analysis

The statistical analysis plan was posted on our website (www.ilins.org) before any analysis was initiated. The primary outcome was maternal total depression score at 6 months postpartum. We also examined the proportion of women who scored ≥12 on the EPDS. All analysis was carried out using SAS for Windows Version 9.4 (SAS Institute, CARY, NC, USA) and was by intention to treat. All participants who had missing data for the outcome variable were excluded from all analysis. Descriptive data are presented as means and standard deviations or frequencies and percentages. Analysis of variance and chi-squared testing were used to compare baseline variables between the three intervention groups. Adherence to supplement intake during pregnancy and lactation was calculated as the percentage of pregnancy days after enrolment to delivery and from delivery to 6 months postpartum, respectively, when supplements were reportedly consumed.

The difference in the depression scores between the three groups was analysed using ANCOVA, with Tukey adjustment for pairwise comparisons. Due to the non-normal distribution of the depression scores, we square-root transformed the variable before use in analysis. The proportion of women who scored ≥12 on the EPDS was compared between the three intervention groups using logistic regression.

We calculated unadjusted means (SD) or group percentages and then repeated the analyses with adjustments for pre-specified covariates. Covariates we examined included maternal years of formal education, height, age, BMI, marital status, anaemia at enrolment, gestational age at enrolment, primiparity, household asset index, household food insecurity index and season when depression data were collected. Only covariates significantly associated with the outcome at 10% level of significance in bivariate analysis were included in the final adjusted model. A *P* value was considered to be significant if *α* <0.05 (unless otherwise stated). We estimated pairwise intervention group differences in means and relative risk for continuous and binary outcomes, respectively.

As per our analysis plan, we examined potential interactions between the intervention group and the variables that could modify the impact of the intervention on depression outcomes. The pre-specified baseline variables include maternal years of formal education, height, age, BMI, marital status, anaemia at enrolment, gestational age at enrolment, primiparity, household asset index, household food insecurity index and season at depression data collection. When an effect modifier was statistically significant (*P* < 0.1), we performed a stratified analysis by assessing the adjusted group effect at different levels of the effect modifier. For a continuous effect modifier, the group effect was compared at the tenth percentile, the median and the 90th percentile for continuous outcomes and for a categorical effect modifier, the group effect was compared for all levels of the effect modifier.

### Ethics

Ethical approval for the study was obtained from the institutional review boards of the University of California, Davis; Ghana Health Service and University of Ghana Noguchi Memorial Institute for Medical Research (NMIMR).

## Results

### Participants

**Fig. 1:**
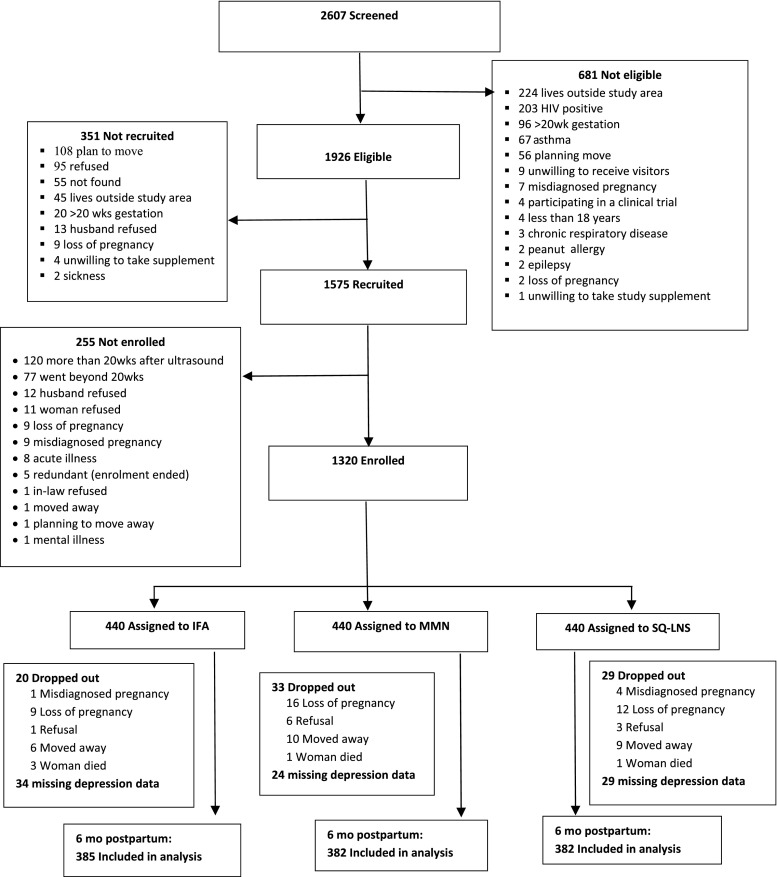
Study profile. The IFA group received 60 mg iron plus 400 mg folic acid. The MMN group received 1–2 Recommended Dietary Allowances of 18 vitamins and minerals (including 20 mg iron). The LNS group received SQ-LNS with the same micronutrients as the MMN group, plus another four minerals (Ca, P, K and Mg), as well as macronutrients. All three supplements were intended for daily consumption. *IFA* iron and folic acid, *SQ-LNS* small-quantity lipid-based nutrient supplement, *MMN* multiple micronutrients

Data were collected between December 2009 and August 2012. Out of 2607 women who were screened at the antenatal visits, 1926 were eligible to be recruited. Of this number, 1575 were recruited (signed consent form) and 1320 enrolled (participated in baseline assessment and randomized to one of the three study groups) (Fig. 1). Of the 1320 enrolled, depression data were available for 87% (LNS = 87%, MMN = 87%, IFA = 88%) and these women were included in this analysis. There were no statistically significant differences in baseline characteristics between participants who were included in this analysis and those who were excluded. The baseline characteristics of women included in the study are shown in Table 2. On average, women were in their mid-twenties, were about 159 cm tall, had about 7 years of formal education and had gestational age of 16 weeks. Almost all women reported being married or cohabiting with a partner. There were no significant differences in baseline characteristics between the three intervention groups within this analytical sample. Reported adherence (percentage of days supplements was reportedly consumed) to supplement intake in women was lower in the LNS compared to the IFA and MMN groups during pregnancy (72.5% ± 21.5 vs 77.4% ± 16.9 vs 76.7% ± 17.2%, respectively, *P* < 0.001) and postpartum (70.0% ± 24.6 vs 77.3% ± 19.6 vs 74.7% ± 21.6%, respectively, *P* < 0.001). As previously reported (Adu-Afarwuah et al. 2015, 2016), we found no significant differences in reported severe adverse events between groups.

**Table 1:** Composition and formulation of supplements used in the study

Nutrient	Chemical form used in supplement formulation	IFA	MMN	SQ-LNS^a^
Ration (g/day)		1 tablet	1 tablet	20
Total energy (kcal)			0	118
Protein (g)			0	2.6
Fat (g)			0	10
Linoleic acid (g)			0	4.59
α-Linolenic acid (g)			0	0.59
Vitamin A (μg RE)	Retinyl acetate		800	800
Vitamin C (mg)	l-ascorbic acid		100	100
Vitamin B_1_(mg)	Thiamin hydrochloride		2.8	2.8
Vitamin B_2_ (mg)	Riboflavin		2.8	2.8
Niacin (mg)	Niacinamide		36	36
Folic acid (μg)	Pteroyl monoglutamic acid	400	400	400
Pantothenic acid (mg)	Calcium pantothenate		7	7
Vitamin B_6_ (mg)	Pyridoxine hydrochloride		3.8	3.8
Vitamin B_12_ (μg)	Cyanocobalamin (0.1%)		5.2	5.2
Vitamin D (IU)	Cholecalciferol (D3)		400	400
Vitamin E (mg)	dl-alpha-tocopherol acetate		20	20
Vitamin K (μg)	Phylloquinone 5%		45	45
Iron (mg)	Encapsulated ferrous sulphate	60	20	20
Zinc (mg)	Zinc sulphate		30	30
Copper (mg)	Encapsulated copper sulphate		4	4
Calcium (mg)	Tricalcium phosphate		0	280
Phosphorus (mg)	Tricalcium phosphate		0	190
	Dipotassium phosphate			
Potassium (mg)	Potassium chloride		0	200
	Dipotassium phosphate			
Magnesium (mg)	Magnesium citrate		0	65
Selenium (μg)	Sodium selenite 1.5%		130	130
Iodine (μg)	Potassium iodate		250	250
Manganese (mg)	Manganese sulphate		2.6	2.6

Information from table previously published from Adu-Afarwuah et al. (2015) and Arimond et al. (2013)

*IFA* iron and folic acid capsule, *MMN* multiple micronutrient supplement capsule, *SQ-LNS* small-quantity lipid-based nutrient supplement for pregnant and lactating women

^a^Nutrient concentrations include contributions from the ingredients as well as from the multiple micronutrient premix

**Table 2:** Baseline characteristics of women by intervention group, among those with data on depression at 6 months postpartum

Variable	LNS (*n* = 382)^a^	IFA (*n* = 385)^a^	MMN (*n* = 382)^a^
Age (years)	27.1 ± 5.5	26.6 ± 5.5	26.8 ± 5.3
Height (cm)	159.1 ± 5.4	159 ± 5.7	159 ± 6.1
BMI (kg/m^2^)	25.2 ± 4.7	24.7 ± 4.5	24.8 ± 4.4
Years of formal education (years)	7.6 ± 3.9	7.8 ± 3.5	7.5 ± 3.6
Married or cohabiting	352 (92.1)	357 (92.7)	361 (94.5)
Primiparous	122 (31.9)	133 (34.5)	123 (32.2)
Anaemia (g/L)^b^	148 (38.7)	164 (42.6)	154 (40.3)
Gestational age at enrolment (weeks)	16.2 ± 3.3	16.0 ± 3.2	16.3 ± 3.2
Season when depression data collected = dry season	185 (48.4)	192 (49.9)	200 (52.4)
Child sex = male	190 (49.9)	175 (45.6)	191 (50.0)
Household asset index	−0.06 ± 1.01	0.03 ± 1.02	0.07 ± 0.94
Housing index	−0.02 ± 1.00	0.02 ± 1.02	−0.02 ± 1.02
Household food insecurity index	2.4 ± 4.0	2.6 ± 4.4	2.7 ± 4.4

LNS group: women were intended to receive 20 g SQ-LNS with same micronutrients as the MMN group, plus another four minerals (Ca, P, K and Mg) as well as macronutrients during pregnancy and the first 6 months postpartum; IFA group: women were intended to receive 60 mg iron plus 400 μg folic acid during pregnancy and a calcium placebo during the first 6 months postpartum; MMN group: women were intended to receive 1–2 RDAs of 18 vitamins and minerals (including 20 mg iron) during pregnancy and the first 6 months postpartum. All three supplements were intended for daily consumption

^a^Values are mean ± SD or *n* (%)

^b^Anaemia based on WHO cut off of 110 g/L

### Main group comparisons

Tables 3 and 4 present the unadjusted and adjusted results for continuous and binary outcomes, respectively, by the intervention group. The reported depression scores at 6 months postpartum did not differ significantly between the three intervention groups (Table 3). Similarly, when we examined the proportion of women who scored ≥12 on the EPDS, we found no significant differences between LNS, IFA and MMN groups (Table 4). Adjustment for covariates did not alter these findings.

**Table 3:** EPDS scores at 6 months postpartum by intervention group (unadjusted and adjusted analysis)

	LNS	IFA	MMN		Comparison between IFA vs LNS		Comparison between MMN vs LNS		Comparison between IFA vs MMN	
	Median (Q1–Q3)^a^	Median (Q1–Q3)	Median (Q1–Q3)	*P* value^b^	Difference (95% CI)	*P* value	Difference (95% CI)	*P* value	Difference (95% CI)	*P* value
EPDS score (unadjusted)	4.0 (1.0–8.0)	4.0 (1.0–8.0)	5.0 (2.0–9.0)	0.13	0.03 (−0.17, 0.24)	0.92	0.17 (−0.04, 0.37)	0.14	−0.13 (−0.34, 0.07)	0.28
EPDS score (adjusted)^c^				0.17	0.02 (−0.18, 0.22)	0.97	0.15 (−0.05, 0.35)	0.19	−0.13 (−0.33, 0.07)	0.29

LNS group: women were intended to receive 20 g SQ-LNS with same micronutrients as the MMN group, plus another four minerals (Ca, P, K and Mg) as well as macronutrients during pregnancy and the first 6 months postpartum; IFA group: women were intended to receive 60 mg iron plus 400 μg folic acid during pregnancy and a calcium placebo during the first 6 months postpartum; MMN group: women were intended to receive 1–2 RDAs of 18 vitamins and minerals (including 20 mg iron) during pregnancy and the first 6 months postpartum. All three supplements were intended for daily consumption

*EPDS* Edinburgh Postnatal Depression Scale

^a^Median (25–75 percentile)

^b^The EPDS scores were square-root transformed for the analysis. Reported *P* values are for the transformed scores

^c^Adjusted for household food insecurity index

**Table 4:** EPDS ≥12 at 6 months postpartum by intervention group (unadjusted and adjusted analysis)

	LNS	IFA	MMN	*P* value	Comparison between IFA vs LNS		Comparison between MMN vs LNS		Comparison between IFA vs MMN	
	*n* (%)	*n* (%)	*n* (%)		Odds ratio (95% CI)	*P* value	Odds ratio (95% CI)	*P* value	Odds ratio (95% CI)	*P* value
EPDS ≥12 (unadjusted)	50 (13.1)	43 (11.2)	56 (14.7)	0.35	0.83 (0.50–1.40)	0.69	1.14 (0.70–1.87)	0.80	0.73 (0.44–1.22)	0.32
EPDS ≥12 (adjusted)^a^				0.32	0.79 (0.47–1.35)	0.55	1.09 (0.67–1.81)	0.89	0.72 (0.43–1.21)	0.30

LNS group: women were intended to receive 20 g SQ-LNS with same micronutrients as the MMN group, plus another four minerals (Ca, P, K and Mg) as well as macronutrients during pregnancy and the first 6 months postpartum; IFA group: women were intended to receive 60 mg iron plus 400 μg folic acid during pregnancy and a calcium placebo during the first 6 months postpartum; MMN group: women were intended to receive 1–2 RDAs of 18 vitamins and minerals (including 20 mg iron) during pregnancy and the first 6 months postpartum. All three supplements were intended for daily consumption

*EPDS* Edinburgh Postnatal Depression Scale

^a^Adjusted for maternal years of education and household food insecurity index

### Effect modification

We examined 11 pre-specified potential effect modifiers, and 3 interactions were significant (*P* < 0.1): season when depression data were collected (*P* = 0.034), housing index (*P* = 0.053) and child sex (*P* = 0.099). Among women interviewed during the dry season, those in the LNS group had lower EPDS scores compared to those in the IFA and MMN groups (1.73 vs 1.95 vs 2.08, *P* = 0.015). By contrast, there were no significant differences between LNS, IFA and MMN groups among women who were interviewed during the wet season (2.07 vs 1.91 vs 2.03, *P* = 0.36). Among women who delivered female infants, there was a trend for those in the LNS group to have lower EPDS scores compared to those in the IFA and MMN groups (1.83 vs 2.01 vs 2.13, *P* = 0.05), but there were no significant differences among women who delivered male infants. Among women who had higher (90th percentile) housing index, those in the IFA group had lower scores compared to the MMN group (*P* = 0.013) but not compared to the LNS group; none of the group differences was significant among women who had a lower housing index.

### Sensitivity analysis

In order to address the protocol violation associated with the consumption of mislabeled capsules by some of the women during pregnancy as described in the methods section above, we conducted two sensitivity analyses that examined data (1) based on the supplement that women actually received when they were enrolled and (2) only for women who received supplements after the mislabeling of IFA and MMN supplements had been corrected.

When we examined the data based on the supplement that women actually received when they were enrolled, we found that, overall, the IFA group recorded the highest depression score compared to LNS and MMN groups in both unadjusted (2.10 vs 1.91 vs 1.90, *P* = 0.039) and adjusted (2.09 vs 1.89 vs 1.91, *P* = 0.032) analysis. In the pairwise comparisons, the IFA group tended to report higher scores compared to the MMN (unadjusted: *P* = 0.07; adjusted: *P* = 0.05) and LNS (unadjusted: *P* = 0.06; adjusted: *P* = 0.07). Depression scores were not significantly different between the MMN and LNS groups in adjusted and unadjusted analysis. The proportion of women who scored ≥12 on the EPDS did not differ significantly between the 3 intervention groups in either adjusted or unadjusted analysis.

In the analysis restricted to women who received supplements after the mislabeling of IFA and MMN supplements had been corrected, we found no significant differences between groups in reported depression scores at 6 months postpartum in either unadjusted or adjusted analysis, or in the proportion of women who scored ≥12 on the EPDS (data not shown).

## Discussion

In this semi-urban area of Ghana where the iLiNS study was conducted, provision of SQ-LNS did not alleviate depressive symptoms at 6 months postpartum. The findings do not support our hypothesis that women who received SQ-LNS during pregnancy would have lower mean EPDS scores at 6 months postpartum compared to women who received either IFA or MMN.

In terms of prevalence of PPD, our results are similar to what was reported in another study in the same study area (9.3%) where our study was conducted (Okronipa et al. 2012). Studies conducted in other African countries have also reported similar rates (11–16%) of PPD (Nhiwatiwa et al. 1998; Stewart et al. 2016; Uwakwe 2003).

Our finding of no impact of SQ-LNS supplementation on maternal PPD is consistent with what was found in the iLiNS-Malawi trial (Stewart et al. 2016) which used the same study design as our study. It is possible that the formulation of SQ-LNS used in these trials (which included 0.59 g alpha-linolenic acid (ALA) and 4.59 g linoleic acid) did not contain enough essential fatty acids (EFA) to influence depression, or there was insufficient conversion of the pre-cursor ALA to the longer chain fatty acids eicosapentaenoic acid (EPA) and docosahexaenoic acid (DHA). A recent meta-analysis of depression trials showed that EPA/DHA supplements containing much higher doses (>60%) of EPA (ranging from 0.2 to 2.2 g, in excess of DHA) were more effective in treating depression (Sublette et al. 2011) than supplements containing lower doses (<60%) of EPA. Thus, increasing the ALA dose in future SQ-LNS supplements to levels higher than we used in our study or including EPA/DHA (with higher EPA dose) may be more likely to influence depression.

Alternatively, it is possible that women in the study area regularly consumed fish with high EFA content and so had adequate EFA status at baseline, making it difficult to observe an effect of SQ-LNS supplementation on depression. Data from the Food and Agriculture Organization balance sheets support the possibility of high fish consumption in Ghana (Michaelsen et al. 2011). Data previously reported from our study also show the EFA status of women at baseline to be within normal levels (Oaks et al. 2017). Additionally, it is also possible that depressive symptoms were not common in our sample at baseline, before supplementation, which would also make it difficult to detect any effect of supplementation. We did not collect data on baseline depression status, so we cannot evaluate this possibility. Lastly, it is possible that other causes of depression were more influential in our population than nutrition-related factors. A number of non-dietary factors including marital status and relationship, social support, childcare stress, infant temperament, socioeconomic status and unplanned pregnancy have been reported to be strong predictors of postpartum depression (Beck 2001).

Results of our pre-specified tests for interactions suggested that season when depression data were collected, child sex and housing index modified the effect of the intervention. Among women interviewed during the dry season, those in the LNS group had significantly lower EPDS scores compared to those in the MMN group. It is possible that SQ-LNS mitigated the impact of the dry season on women’s depressive symptoms. The dry season is usually the post-harvest season, the period before the next planting season when food is sometimes scarce and food insecurity may be common. Similarly, among women who delivered female infants, those in the LNS group tended to have lower depression scores compared to those in the MMN group. Studies in India (Patel et al. 2002), Nigeria (Adewuya et al. 2005) and Turkey (Ekuklu et al. 2004) have reported PPD to be more common among mothers of female babies, and this has usually been explained by the societal preference for male compared to female children. Nonetheless, we cannot completely rule out the possibility that the interaction results may be spurious.

Strengths of our study include the randomized design of the trial, blinding of study participants to IFA/MMN treatment, large sample size, long follow-up and low drop-out rate. There were a few limitations that warrant mention. First, we did not have data on the depression symptoms in our sample at baseline, before supplementation. Second, due to the differences between the IFA/MMN capsules and SQ-LNS sachets, we were unable to blind study participants and field workers to IFA/MMN vs SQ-LNS treatment. However, field workers were blinded to the study hypothesis and taken through regular refresher training, and data analysts were fully blinded to group assignments until analyses were completed. Thirdly, in spite of our effort to ensure good quality data by organizing regular refresher training for our data collectors, the possibility of measurement error still remains, which could also have influenced our results and estimate of depression in the Ghanaian population. Lastly, in this study, we measured depression using a screening tool instead of a diagnostic interview. We acknowledge that using a diagnostic interview would have increased our confidence in our results, but we were limited by resource constraints. It is worth noting, however, that the EPDS screening tool has been widely validated and used in different low-income populations including Ghana (Nhiwatiwa et al. 1998; Okronipa et al. 2012; Stewart et al. 2016; Uwakwe 2003) to measure symptoms of depression. We therefore believe that the study’s weaknesses do not bias the finding of no association of maternal SQ-LNS consumption during pregnancy and lactation with depressive symptoms at 6 months postpartum.

In conclusion, this study did not provide evidence to support the hypothesis that supplementing maternal diet with SQ-LNS will improve maternal depression scores at 6 months postpartum. However, the significant interaction with season warrants further study.
